# Effect of maternal pre-pregnancy underweight and average gestational weight gain on physical growth and intellectual development of early school-aged children

**DOI:** 10.1038/s41598-018-30514-6

**Published:** 2018-08-13

**Authors:** Chao Li, Ni Zhu, Lingxia Zeng, Shaonong Dang, Jing Zhou, Leilei Pei, Victoria Watson, Tao Chen, Duolao Wang, Hong Yan

**Affiliations:** 1 Department of Epidemiology and Biostatistics, School of Public Health, Xi’an Jiaotong University Health Science Center, Xi’an, China; 2 Department of Health Information, Shaanxi Provincial Center for Disease Control and Prevention, Xi’an, China; 3 Department of Clinical Sciences, Liverpool School of Tropical Medicine, Pembroke Place, Liverpool, United Kingdom; 4 Nutrition and Food Safety Engineering Research Center of Shaanxi Province, Xi’an, China; 5 Key Laboratory of Environment and Genes Related to Diseases, Xi’an Jiaotong University, Xi’an, China

## Abstract

The aim of this study was to assess the effect of low maternal weight at pre-pregnancy and the average gestational weight gain on undernourished children and their intellectual development. From October 2012 to September 2013, we followed 1744 offspring of women who participated in a trial conducted from 2002 to 2006. Pregnant women recruited in the original trial could receive three prenatal health checks for free, at which maternal weight and height were measured. WISC-IV was used to estimate the intellectual development of children. Weight and height of both pregnant women and children were measured by trained anthropometrists using standard procedures. Having low maternal weight at pre-pregnancy was associated with an increased risk of undernutrition amongst children (underweight: OR = 2.02, 95%CI: 1.14–3.56, thinness: OR = 2.79, 95%CI: 1.50–5.17) and a decrease in verbal comprehension index (−2.70 points, 95%CI: −4.95–0.44) of children. The effect of average gestational weight gain on occurrences of underweight children (OR = 0.08, 95%CI: 0.01–0.55) was also found. We identified the effect of maternal pre-pregnancy underweight on impairment of the separate intellectual domains (verbal comprehension index) and increasing occurrence of undernourished children. Average gestational weight gain was positively associated with a decreased prevalence of underweight children but not with the intellectual development of children in rural China.

## Introduction

Maternal nutrition before and during pregnancy is recognized as being an important factor for offspring health^1^. Although a number of studies in developed countries focused on the association between high maternal pre-pregnancy BMI (overweight or obesity) and offspring obesity and intellectual development^2–4^, studies on pre-pregnancy underweight and associated outcomes are rarely studied. In many developing countries maternal underweight was more common than maternal overweight^5^. Recent research suggests that offspring of underweight mothers may also have impaired intellectual development. Maternal underweight is a known risk factor for low birth weight which is predictor of adverse brain development^6,7^. In addition, evidence from the US cohort study showed low pre-pregnancy BMI was associated with increased risk of delayed intellectual development of 2-year-old children^8^. To the best of our knowledge, the effect of maternal underweight on offspring occurrence of stunting, thinness and underweight in childhood was rarely reported in developing countries.

Although previous results showed that gestational weight gain is associated with an increased BMI of offspring in childhood, adolescence and early adulthood^9–12^, few studies reported the effect of gestational weight gain on the risk of undernutrition in childhood. The data examining the association between gestational weight gain and child intellectual development is limited^13–15^. Results are also inconsistent, with a large European cohort study reporting a decrease in offspring intelligence with increasing gestational weight gain^13^, whereas a null finding on the relation between gestational weight gain and offspring intelligence was reported in 2 studies^14,15^.

For childhood physical growth, many studies also determined the negative effect of malnutrition on their motor, cognitive and social-emotional development^7,16,17^. For childhood intellectual development, results from a series of population-based prospective cohort studies indicated that childhood intelligence quotient (IQ) is associated with further leadership success and school achievement and is inversely associated with several health outcomes ascertained in later life^18–20^. Therefore, understanding the effect of maternal BMI and gestational weight gain on offspring physical and intellectual development is vital in developing countries^10^. The aim of this study is to clarify the effect of low pre-pregnancy weight and gestational weight gain on physical and intellectual development at early school-aged children. The study field was rural areas of China and the type four villages, the poorest. Therefore, results from this study could provide important reference for improving physical development and intellectual development at early school-aged children in poor areas of developing countries.

## Methods

### Study design and participants

The present follow-up study was conducted from October 2012 to September 2013. We followed the offspring of women who had participated in the large trial of prenatal micronutrient supplementation and remained residents in the original study area. The baseline information was obtained from this trial, and the results of this trial are described elsewhere^21–23^. In summary, this was a double-blind, cluster-randomized, controlled trial of prenatal supplementation with three different combinations of micronutrients implemented from 2002 to 2006 that aimed to determine the effect of prenatal micronutrient supplementation on birth weight in two rural counties in Shaanxi Province of Northwest China. The enrolled pregnant women in the same village were randomly assigned to 3 supplementation groups (daily folic acid, folic acid plus iron, or multi-micronutrients), and all pregnant women in the same village were allocated to the same treatment. To ensure geographic balance, villages were also stratified according to township and county. Finally, 5828 pregnant women from 531 villages enrolled, and there were 4604 single live births^21–23^.

**Figure 1:**
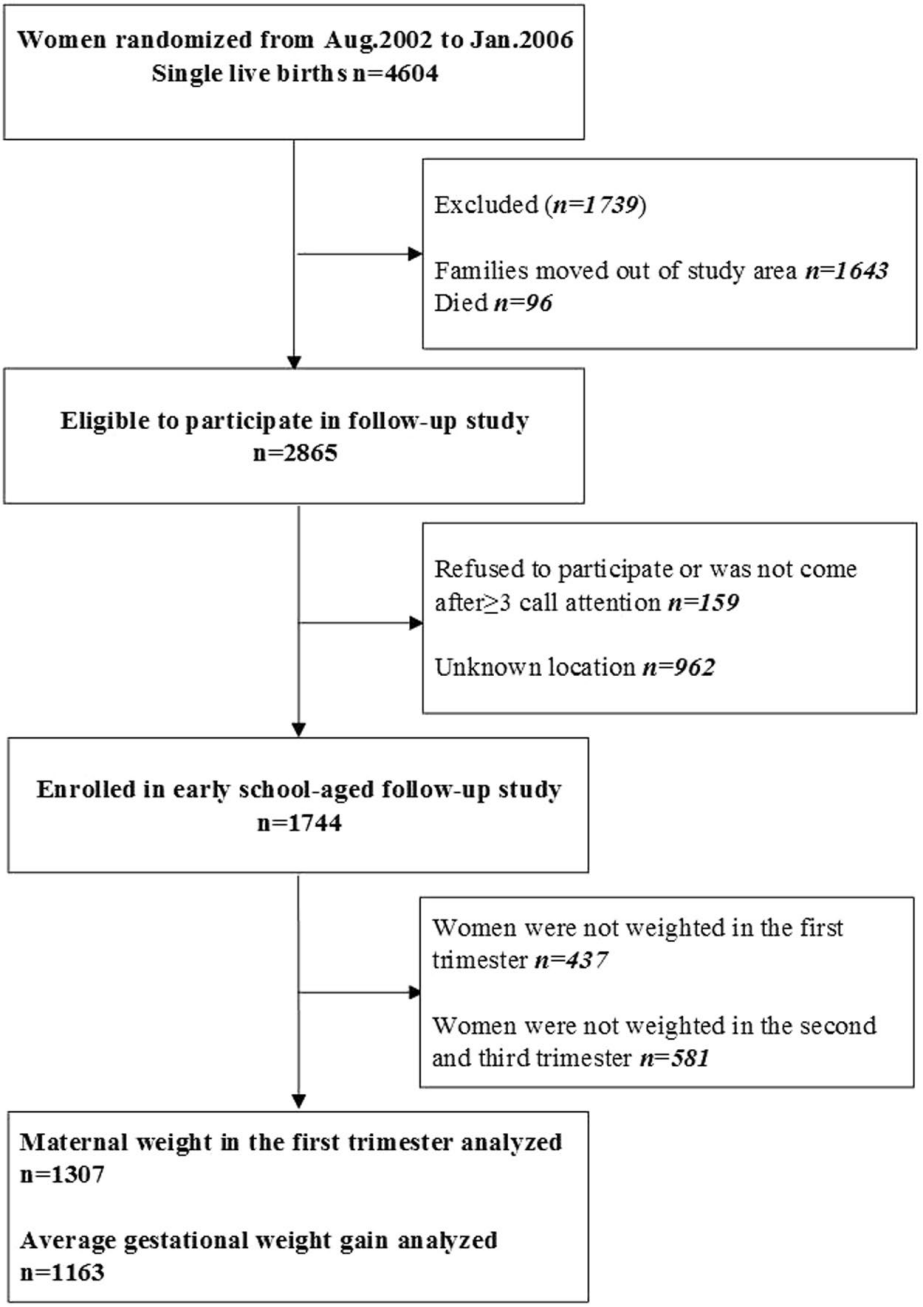
Participant flow chart.

In the present follow-up study, we excluded migrations because of the limitation of funding and it was not possible to trace them. More importantly, the migrations were not representative of rural China. Households with eligible children were invited to participate in the local hospital or school in a standardized manner. Parental written informed consent and child assent were obtained. By September 2013, 96 children had died, and 1643 children had moved out of the study area with their family. Of the remaining 2865 children eligible for inclusion in present study, we followed 1744 of these eligible children (159 participates refused to take part or were not able to come after ≥3 reminders; 962 unknown location). Our previous published results reported participants and those who did not take part in the early school-aged follow-up study did not differ in any enrollment measure^22^. From this group, we also excluded women with missing information about important variables, i.e., pre-pregnancy BMI (n = 437), and average gestational weight gain (n = 581) (Fig. 1).

This follow-up study was designed to estimate the intellectual function differences among prenatal micronutrients supplementation groups, a minimum of 426 children (142 in each group) was needed to detect 5 Full-Scale intelligence Quotient (FSIQ) points between groups with type I error and 80% power. Sample size in the present study (n = 1307) was large enough to detect 5 FSIQ points between maternal pre-pregnancy underweight and normal weight group. According to the results of previous studies, 5-IQ point difference was considered clinically significant because it is of the order of magnitude associated with IQ differences in children who were exposed to high lead concentrations or were fed breast milk rather than formula as infants^24,25^. The study was conducted in accordance with the declaration of Helsinki and was approved by the Human Research Ethics Committee of the Xi’an Jiaotong University Health Science Center.

## Measurements

### Maternal pre-pregnancy BMI and gestational weight gain

Maternal BMI during the first trimester is being used as a proxy for pre-pregnancy BMI As most women, especially in the undernourished populations, do not gain much weight in the first trimester (usually only a couple kilograms in US populations) and self-reported pre-pregnancy weights are often misreported. Findings from a Caucasian study indicate mean maternal weight did not change at all in the first trimester^26^. Pre-pregnancy BMI was categorized as underweight (BMI < 18.5), normal-weight (18.5 ≤ BMI < 25), overweight (25 ≤ BMI < 30), or obese (BMI ≥ 30)^27^. Pregnant women recruited in the original large trial could receive three prenatal health checks for free, at which maternal weight and height were measured at the clinic by trained maternal and child health (MCH) staff. In addition, pregnant women were interviewed to record their sociodemographic status such as educational levels, occupations of parents, and the number of older siblings and their menstrual, reproductive, medical, and family history. The total gestational weight gain was calculated from the weight measured in the third trimester (the last prenatal health check) and maternal weight measured in the first trimester (0–14 weeks of gestational age). Gestational age used to calculate weekly gestational weight gain was calculated from the weeks of gestation in the last prenatal health check in the third trimester minus weeks of gestation in the first trimester. Weekly gestational weight gain is calculated from total gestational weight gain divided by gestational age. Gestational age at birth was measured as completed days based on the first day of the last menstrual period.

### Physical growth

The physical growth was estimated by weight and height of children in the present follow-up study. The anthropometric measurements of early school-aged children were measured using standard procedures by trained staff. Weight was recorded in standard school clothing (without shoes) using an electronic scale (Tanita BC-420, Tanita Corporation, Tokyo, Japan) with precision to the nearest 10 g, and height (barefooted) was recorded using a calibrated stadiometer (Model SZG-210, Shanghai JWFU Medical Apparatus Factory, Shanghai, China) with precision to the nearest 0.1 cm. Weight-for-age z-scores, height-for-age z-scores (HAZ), and body mass index-for-age z-scores (BAZ) were derived from these observational data based upon 2007 ref.^28^. Stunting, thinness and underweight children were defined as HAZ, BAZ and WAZ of ≤−2, respectively.

### Intellectual development

The fourth edition of Wechsler intelligence scale for children (WISC-IV) which can be applied to children aged 6 to 16 years is not only among the most widely used intelligence tests in the world, but also the latest edition of Wechsler intelligence scale for children. In the present study, WISC-IV which has been commercialized and standardized to be culturally appropriate in China, was used to evaluate the intellectual development of early school-aged children. Currently, the reliability and validity of these measures were evaluated and shown to be satisfactory^29^. There are 10 core subtests (Block Design, Similarities, Comprehension, Vocabulary, Picture Concepts, Digit Span, Letter–Number Sequence, Matrix Reasoning, Coding and Symbol Search) and 4 supplemental subtests (Picture Completion, Information, Cancellation and Arithmetic) included in WISC-IV. In addition, a FSIQ, which represents overall cognitive ability, and 4 other composite scores (verbal comprehension index VCI, working memory index WMI, processing speed index PSI, and perceptual reasoning index PRI) which represent different domains of cognitive function^30^ could been generated from WISC-IV. In more detail, VCI represents the ability in verbal reasoning and concept formation. WMI represents the ability to sustain attention, concentrate, and exert mental control, concentrate. PSI represents the ability in routine visual material without making errors or processing simple. PRI is designed to measure the ability to separate ground and figure in visual stimuli, and fluid reasoning in the perceptual domain^31^.

WISC-IV is a standardized test, with the same scoring criteria and method, and the same order of administered subsets. The intelligence test was done by five postgraduate students in psychology, and they were overseen and rigorously trained by LC who qualified to administer the WISC-IV and served as psychometrician. The five students were certified to collect data when they performed an accurate test administration and scoring. To ensure the children would not be interrupted, all the intelligence tests were conducted in the local hospital or primary school where the hospital or school was asked to provide 5 quiet and separated rooms. After field work was completed each day, the answers of intelligence tests recorded by each student were reviewed by the psychometrician to ensure the scoring accuracy of each child.

### Statistical analysis

All data were checked manually for completeness and double-entered for verification. Range, extremum and logical checks were conducted for accuracy. A 5% significance level was used for all statistical tests, and testing was two-sided. The distributions of baseline information in different groups of maternal pre-pregnancy nutritional status were described by their means, standard deviations, and percentages. In addition, the ownership of 16 different household facilities or assets were used to construct a household wealth index using principal components method to assign a weight for each assets^32^, and the household wealth index was categorized as tertiles indicating the poorest, middle and richest households.

In the present study, multilevel mixed-effects generalized linear models (township to level 3, village to level 2, and individual to level 1) were used to analyze the effect of maternal pre-pregnancy nutritional status and average gestational weight gain on intellectual development (WISC-IV tests scores) and malnutrition status (underweight, stunting and thinness) of early school-aged children, and the effects were reported in terms of the estimations of coefficients and 95% confidence intervals (CIs). As maternal overweight and obesity is associated with risk of several diseases in offspring, the main results were reported after excluding overweight and obesity participants. Previous studies identified age of children, sex of children, gestational weeks, economic status, prenatal micronutrients supplementation, birthweight and other social backgrounds as detrimentally affecting cognitive development of children^22,28,33,34^. In addition, the majority of variables mentioned above were also reported as being associated with physical growth of children in previous studies^35–37^. Therefore, we considered the following variables as potential confounders in the multivariate adjusted analysis: age of children, age of parents, sex of children, gestational weeks at birth, type of micronutrient supplement, household wealth index, older siblings of children, birthweight, educational level of parents, father’s occupation of parents, child school level.

To test the reliability of the analysis, two models were fitted with one model adding variables of social backgrounds (the age of children, age of parents, sex of children, educational level of parents, occupation of parents, household wealth index), and the other model including the variables of social backgrounds plus the variables of older siblings of children, child school level, gestational weeks at birth, weight at birth, type of prenatal micronutrient supplementation. When we estimated the effects of gestational weight gain, an additional variable of maternal BMI at pre-pregnancy was considered. Stata software 14.0 was used for all data analysis (Stata Corp LP, College Station, Texas 77845, USA).

## Results

Table 1 shows the baseline characteristics of children and their households in different groups of maternal pre-pregnancy nutritional status. The prevalence of maternal overnutrition (overweight or obesity) and those underweight was 2.5% (overweight: 32/1307; obesity: 1/1307) and 15.4% (201/1307) respectively. For the characteristics of children, the mean age was 8.78 (SD, 0.82) years; the majority of children (40.1%) were studied in schools of township level; mean FSIQ, VCI, WMI, PRI and PSI of investigated children were 89.58, 88.02, 91.36, 93.19 and 95.71 respectively, and WISC-IV tests scores of children were higher in the maternal non-underweight group, except for PRI and PSI. For nutritional status of children, the prevalence of those underweight (11.0%), stunting (6.0%) and thinness (12.4%) were higher in maternal underweight group compared with 6.1%, 3.5% and 5.5% in non-underweight group. Average gestational weight gain (0.36 kg/wk) was higher in the maternal underweight group than maternal non-underweight group (0.33 kg/wk).

**Table 1:** Baseline characteristics of children and households by pre-pregnancy nutritional status^a^.

	Maternal pre-pregnancy BMI		Total
	Underweight	Non-underweight	
	201	1106	1307
**Child characteristics**			
Child age, mean ± SD, y	8.83 ± 0.81	8.77 ± 0.83	8.78 ± 0.82
School, n (%)			
Village	68(33.8)	380(34.4)	448(34.3)
Township	66(32.9)	458(41.4)	524(40.1)
County	67(33.3)	268(24.2)	335(25.6)
Birth weight, mean ± SD, kg	3.09 ± 0.44	3.22 ± 0.43	3.20 ± 0.43
Gestational age at birth, mean ± SD, wk	39.69 ± 1.53	39.84 ± 1.66	39.82 ± 1.64
FSIQ, mean ± SD	89.22 ± 14.87	89.65 ± 13.13	89.58 ± 13.41
VCI, mean ± SD	86.39 ± 15.60	88.31 ± 15.78	88.02 ± 15.76
WMI, mean ± SD	90.99 ± 13.06	91.43 ± 12.24	91.36 ± 12.36
PRI, mean ± SD	93.98 ± 14.30	93.05 ± 13.62	93.19 ± 13.73
PSI, mean ± SD	96.48 ± 14.81	95.57 ± 12.73	95.71 ± 13.06
Weight for age			
Underweight	22(11.0)	67(6.1)	89(6.8)
Non-underweight	179(89.0)	1039(93.9)	1218(93.2)
Height for age			
Stunting	12(6.0)	39(3.5)	51(3.9)
Non-stunting	189(94.0)	1066(96.5)	1255(96.1)
BMI for age			
Thinness	25(12.4)	61(5.5)	86(6.6)
Non-thinness	176(87.6)	1043(94.5)	1219(93.4)
**Women’s characteristics**			
Maternal age, mean ± SD, y	33.06 ± 4.20	34.14 ± 4.57	33.98 ± 4.53
Parity, n (%)			
0	146(72.6)	674(60.9)	820(62.7)
1	50(24.9)	371(33.6)	421(32.2)
≥2	5(2.5)	61(5.5)	66(5.1)
Women’s education, n (%)			
Primary	68(33.8)	413(37.4)	481(36.8)
Secondary	98(48.8)	564(51.0)	662(50.7)
≥High school	35(17.4)	128(11.6)	163(12.5)
Women’s occupation at enrollment, n (%)			
Farmer	122(61.6)	729(67.2)	851(66.4)
Others	76(38.4)	355(32.8)	431(33.6)
Average gestational weight gain, mean ± SD, kg/wk	0.36 ± 0.21	0.33 ± 0.16	0.33 ± 0.17
**Others**			
Type of prenatal micronutrients supplementation, n (%)			
Folic acid	70(34.8)	363(32.8)	433(33.1)
Iron/folic acid	71(35.3)	372(33.6)	443(33.9)
Multimicronutrients	60(29.9)	371(33.6)	431(33.0)
Maternal age, mean ± SD, y	35.86 ± 4.36	36.67 ± 4.50	36.54 ± 4.49
Father’s education, n (%)			
Primary	32(16.0)	190(17.2)	222(17.0)
Secondary	120(60.0)	685(62.1)	805(61.7)
≥High school	48(24.0)	229(20.7)	277(21.3)
Father’s occupation at enrollment, n (%)			
Farmer	55(27.8)	414(38.2)	469(36.6)
Others	143(72.2)	671(61.8)	814(63.4)
Wealth index at enrollment, n (%)			
Poorest	58(28.9)	356(32.2)	414(31.7)
Middle	66(32.9)	370(33.4)	436(33.3)
Richest	77(38.3)	380(34.4)	457(35.0)

Abbreviation: FSIQ, Full-Scale Intelligence Quotient; PRI, Perceptual Reasoning Index; PSI, Processing Speed Index; VCI, Verbal Comprehension Index; WMI, Working Memory Index.

^a^Values are n(%) or means ± SDs.

Table 2 shows the associations between maternal pre-pregnancy nutritional status, average gestational weight gain and malnutrition status of early school-aged children. After adjusting for the confounders, the effect of low maternal weight on an underweight childhood (Model 1: OR = 1.99, 95%CI: 1.14–3.49; Model 2: OR = 1.95, 95%CI: 1.11–3.45) and thinness (Model 1: OR = 2.91, 95%CI: 1.62–5.23; Model 2: OR = 2.65, 95%CI: 1.43–4.92) remained significant. A negative association between average gestational weight gain and childhood underweight was found (OR = 0.09, 95%CI: 0.02–0.53) after controlling for the confounders (model 1). Results from model 2 suggested every 1 kg/wk increasing of average gestational weight gain, the odds of being underweight will decrease 0.08 (95% CI: 0.01–0.55) times.

**Table 2:** Association between maternal pre-pregnancy nutritional status, average gestational weight gain and malnutrition status of early school-aged children^a^.

	Underweight of children		Stunting of children		Thinness of children	
	OR(95%CI)	*P*	OR(95%CI)	*P*	OR(95%CI)	*P*
**Unadjusted analysis**						
Maternal pre-pregnancy nutritional status						
Underweight	2.00(1.18,3.37)	0.010	1.82(0.90,3.67)	0.094	2.61(1.50,4.54)	0.001
Normal	1.00		1.00		1.00	
Average gestational weight gain, kg/wk	0.08(0.02,0.43)	0.003	0.50(0.07,3.43)	0.482	1.26(0.32,4.95)	0.744
**Model 1** ^**b**^						
Maternal pre-pregnancy nutritional status						
Underweight	1.99(1.14,3.49)	0.016	1.95(0.92,4.11)	0.080	2.91(1.62,5.23)	<0.001
Normal	1.00		1.00		1.00	
Average gestational weight gain, kg/wk	0.09(0.02,0.53)	0.008	0.64(0.09,4.44)	0.652	1.65(0.43,6.35)	0.465
**Model 2** ^**c**^						
Maternal pre-pregnancy nutritional status^d^						
Underweight	1.95(1.11,3.45)	0.021	1.16(0.53,2.57)	0.712	2.65(1.43,4.92)	0.002
Normal	1.00		1.00		1.00	
Average gestational weight gain, kg/wk	0.08(0.01,0.55)	0.010	0.87(0.14,5.35)	0.880	1.37(0.32,5.82)	0.670

Abbreviation: CI, Confidence interval.

^a^Multilevel models were used to assess the association between pre-pregnancy nutritional status, average gestational weight gain and physical growth in early school aged children, with township to level 3, village to level 2, and individual to level 1 after excluding overweight and obesity participants.

^b^Model 1 included the variables of county, the age of children, parental age, sex of children, educational level of parents, occupation of parents, household wealth index.

^c^Model 2 included the variables of county, the age of children, parental age, sex of children, educational level of parents, occupation of parents, household wealth index, older siblings of children, child school level, gestational weeks at birth, weight at birth, type of prenatal micronutrient supplementation.

^d^When estimate the effects of gestational weight gain, one more variable of maternal BMI at pre-pregnancy is added into the mode 2.

Table 3 shows the effects of low maternal weight at pre-pregnancy on VCI scores. After adjusting for confounders, we found the VCI score (Model 1: 2.75 points, 95%CI: −4.99–0.50; Model 2: 2.70 points, 95%CI: −4.95–0.44) was significantly lower in the maternal underweight group compared with the non-underweight group. However the effects of low maternal weight at pre-pregnancy was not found on FSIQ (Model 2: −0.76 points, 95%CI: −2.60–1.08), WMI (Model 2: −0.07 points, 95%CI: −1.85–1.71), PRI (Model 2: 0.84 points, 95%CI: −1.12–2.79) and PSI Model 2: 1.43 points, 95%CI: −0.47–3.34) scores.

**Table 3:** Association between pre-pregnancy nutritional status, average gestational weight gain and intellectual development of early school-aged children^a^.

	Underweight of women		Average gestational weight gain^d^	
	MD(95%CI)	*P*	Coef(95%CI)	*P*
**Unadjusted analysis**				
FSIQ	−1.18(−3.12,0.75)	0.231	2.71(−1.77,7.18)	0.236
VCI	−2.89(−5.16,−0.63)	0.012	5.22(−0.04,10.47)	0.052
WMI	−0.72(−2.54,1.10)	0.439	1.18(−3.00,5.35)	0.581
PRI	0.18(−1.86,2.23)	0.859	0.15(−4.49,4.79)	0.950
PSI	0.47(−1.46,2.41)	0.632	1.99(−2.41,6.39)	0.375
**Model 1** ^**b**^				
FSIQ	−1.11(−2.95,0.72)	0.235	2.27(−1.98,6.51)	0.238
VCI	−3.08(−5.24,−0.92)	0.005	3.85(−1.14,8.84)	0.130
WMI	−0.35(−2.12,1.43)	0.700	1.35(−2.68,5.39)	0.511
PRI	0.19(−1.78,2.17)	0.849	0.35(−4.11,4.82)	0.877
PSI	0.76(−1.14,2.67)	0.435	2.07(−2.19,6.33)	0.341
**Model 2** ^**c**^				
FSIQ	−0.76(−2.60,1.08)	0.418	2.34(−1.94,6.62)	0.284
VCI	−3.14(−5.30,−0.98)	0.004	4.24(−0.78,9.26)	0.098
WMI	−0.07(−1.85,1.71)	0.937	1.14(−2.94,5.22)	0.585
PRI	0.84(−1.12,2.79)	0.402	0.05(−4.41,4.51)	0.983
PSI	1.43(−0.47,3.34)	0.140	2.31(−2.00,6.63)	0.294

Abbreviation: CI, Confidence interval. FSIQ, Full-scale Intelligence Quotient. MD, Mean Difference. PRI, Perceptual Reasoning Index. PSI, Processing Speed Index. VCI, Verbal Comprehension Index. WISC-IV, Wechsler Intelligence Scale for Children Fourth Edition; WMI, Working Memory Index.

^a^Multilevel models were used to assess the association between pre-pregnancy nutritional status, average gestational weight gain and intellectual development in early school aged children, with township to level 3, village to level 2, and individual to level 1 after excluding overweight and obesity participants.

^b^Model 1 included the variables of county, the age of children, parental age, sex of children, educational level of parents, occupation of parents, household wealth index.

^c^Model 2 included the variables of county, the age of children, parental age sex of children, educational level of parents, occupation of parents, household wealth index, older siblings of children, child school level, gestational weeks at birth, weight at birth, type of prenatal micronutrient supplementation.

^d^When estimate the effects of gestational weight gain, one more variable of maternal BMI at pre-pregnancy is added into the mode 2.

## Discussion

In this study we found a negative effect of low maternal weight at pre-pregnancy on nutritional status (underweight and thinness) and verbal comprehension in early school-aged children. Average gestational weight gain was positively associated with a decrease in prevalence of underweight children but not with the intellectual development of children.

Results from many previous studies suggest a detrimental influence of low and high pre-pregnancy BMI and gestational weight gain on physical growth and intellectual development^12,38,39^. For physical growth, results from a systematic review showed that high pre-pregnancy BMI was associated with offspring overweight or obesity^2^. Another tow US studies reported that excessive gestational weight gain is significantly associated with the risk of being overweight in the offspring at 5 and 7 years of age^10,11^. To the best of our knowledge, the effect of maternal underweight on the risk of undernutrition in childhood was rarely reported. Another US study observed a U-shaped association between maternal gestational weight gain and child weight outcomes^12^. For intellectual development, results from a birth cohort study showed that low and very-high maternal pre-pregnancy BMI were associated with increased risk of delayed mental development among 2-year-old US children^8^. Another study conducted in the US reported that a detrimental influence of high gestational weight gain on intellectual development at 4 and 7 years of age^14^, but similar associations between low gestational weight gain and intellectual development of children was not found. Another longitudinal study reported a 0.02–0.07 point increase in children’s IQ with increasing trimester specific gestational weight gain^13^. Although in contrast to our results, this 0.02–0.07 increase in IQ is too small to have clinical implications, as previous studies reported that a 5 IQ point difference was considered clinically significant between intervention and control group^24,25^. Our findings are consistent with the remainder of studies as we also determine the negative effect of maternal underweight (low maternal pre-pregnancy BMI) on both physical growth and intellectual development of children, but we were unable to examine the effect of maternal obesity. Due to the target study fields being the poorest rural areas in China, overweight and obesity prevalence was low meaning the sample size was not large enough to analyze the association between gestational weight gain and risk of offspring obesity and being overweight. In addition, we were also unable to evaluate the effect of excessive or inadequate gestational weight gain due to it being calculated as the last weight at the third trimester of pregnancy (not at delivery) minus pre-pregnancy weight, therefore weight gain during the whole pregnancy could not be calculated.

The observed effect of maternal pre-pregnancy underweight on further development may be attributable to several mechanisms and potential biological mechanisms may include inadequate prenatal micronutrient status. Our previous results demonstrated the positive effect of prenatal micronutrients supplementation on increasing birthweight^21^, the mental development of 1-year old infants^23^. These results could provide indirect evidence supporting the possibility that prenatal micronutrients status may be the potential mechanism of these findings. Other hypothesized downstream pathways include gestational weight gain, birthweight, or the postnatal environment^8,40^. With regards to gestational weight gain, weight gains below the required amount for the products of conception have been hypothesized to be associated with delayed development via inadequate fetal growth or maternal ketosis^40^. Our previous results emphasis the importance of prenatal multi-micronutrients on increasing birthweight compared to the control group^21^, and the long-term effect of birthweight was also found on further intellectual development and physical growth^7^. In addition, postnatal nutritional factors, such as protein intake or breastfeeding, may also affect early child development^41,42^, although this pathway could be either biological or behavioral. Nonetheless, further investigations designed to understand the biological mechanisms of this unfavorable association are critically needed.

This present study has several strengths. Firstly, the information such as gestational weeks or birth weight was collected during the time of pregnancy or at birth, ensuring the accuracy and avoiding potential biases in using recalled information. Secondly, because of the nature of longitudinal data, we could reveal linkages between maternal weight in the first trimester, average gestational weight gain and physical growth of children. Finally, a well-known and standardized intelligence scale (WISC-IV) for use in various cultures was used to estimate intellectual development of children in the study. Our study also has several limitations. One limitation was our inability to follow all children in the original cohort. This limitation was mostly caused by rapid development of China, with mass migration out of the original area, making it impossible to trace the participants. After excluding death and migration, we compared the baseline characteristics between follow-up and lost to follow-up participants and we found there was no difference between the two groups in baseline characteristics (Supplementary Table). After excluding participants of migrations, the ability to generalize the population in other areas was limited, yet the data in the present study is still meaningful rare, and representative of rural China. Another limitation was that we could not determine the mechanisms by which low maternal weight may affect children’s physical and intellectual development. Although we discussed the possible mechanism, the present findings of effect on impairment of verbal comprehension index were rarely found, and further similar studies were needed to clarify the mechanisms of effect of maternal underweight at pre-pregnancy on childhood impairment of verbal ability. In addition, we did not include all possible confounders (such as parental intellectual function, diet of children and smoking) in our analysis, mainly because of small number of smokers among mothers and availability of dietary data. For parental intellectual function, a previous study reported it may affect childhood development via feeding practice or physical activity, and it is also reported as a strong factor of intellectual function of offspring^43^. Finally, due to the sample size not being large enough (prevalence of maternal overweight or obesity was only 2.45%, 33/1307), and missing data of maternal weight at delivery, we could not estimate the effect of maternal obesity, inadequate or excessive gestational weight gain. Although there were several limitations in the present study, most similar studies were conducted in developed counties, and focus mainly on those who suffer from being overweight and obese, while largely neglecting research into malnutrition status in poor areas. Present findings can provide guidance for health professionals to further improve children’s physical growth and intellectual development. Similar studies implemented in developing countries were mainly cross-sectional studies, and longitudinal data from prospective cohort studies was rare.

In conclusion, we identified the effect of low maternal pre-pregnancy weight on malnutrition status (underweight and thinness) and the verbal comprehension ability of children at school age. The positive effect of average gestational weight gain on decreasing occurrence of underweight children in poor areas of China was also identified. Our findings reinforce the importance of encouraging women in poor areas to achieve healthy gestational weight gain and weight before pregnancy on further physical and intellectual development of children. Further research is required to explore possible biological mechanisms by which having a low maternal weight may affect children’s physical and intellectual development.

## Electronic supplementary material

Supplementary table 1 Baseline characteristics comparison between follow-up and lost to follow-up groups

## References

[1] Ramakrishnan U, Grant F, Goldenberg T, Zongrone A, Martorell R (2012). Effect of women’s nutrition before and during early pregnancy on maternal and infant outcomes: a systematic review. Paediatric and perinatal epidemiology.

[2] Yu Z (2013). Pre-pregnancy body mass index in relation to infant birth weight and offspring overweight/obesity: a systematic review and meta-analysis. PloS one.

[3] Brion MJ (2010). Intrauterine effects of maternal prepregnancy overweight on child cognition and behavior in 2 cohorts. Pediatrics.

[4] Veena SR (2016). Association between maternal nutritional status in pregnancy and offspring cognitive function during childhood and adolescence; a systematic review. BMC pregnancy and childbirth.

[5] Neggers YH (2015). The relationship between preterm birth and underweight in Asian women. Reproductive Toxicology.

[6] de Kieviet JF, Zoetebier L, Van Elburg RM, Vermeulen RJ, Oosterlaan J (2012). Brain development of very preterm and very low-birthweight children in childhood and adolescence: a meta‐analysis. Developmental Medicine & Child Neurology.

[7] Li C (2016). Effect of prenatal and postnatal malnutrition on intellectual functioning in early school-aged children in rural western China. Medicine.

[8] Hinkle SN (2012). Associations between maternal prepregnancy body mass index and child neurodevelopment at 2 years of age. International Journal of Obesity.

[9] Streuling I (2011). Physical activity and gestational weight gain: a meta‐analysis of intervention trials. BJOG: An International Journal of Obstetrics & Gynaecology.

[10] Hinkle SN (2012). Excess gestational weight gain is associated with child adiposity among mothers with normal and overweight prepregnancy weight status. The Journal of nutrition.

[11] Wrotniak BH, Shults J, Butts S, Stettler N (2008). Gestational weight gain and risk of overweight in the offspring at age 7 y in a multicenter, multiethnic cohort study. The American journal of clinical nutrition.

[12] Oken E, Rifas-Shiman SL, Field AE, Frazier AL, Gillman MW (2008). Maternal gestational weight gain and offspring weight in adolescence. Obstetrics and gynecology.

[13] Gage SH, Lawlor DA, Tilling K, Fraser A (2013). Associations of maternal weight gain in pregnancy with offspring cognition in childhood and adolescence: findings from the Avon Longitudinal Study of Parents and Children. American journal of epidemiology.

[14] Keim SA, Pruitt NT (2012). Gestational weight gain and child cognitive development. International journal of epidemiology.

[15] Tanda R, Salsberry PJ, Reagan PB, Fang MZ (2013). The impact of prepregnancy obesity on children’s cognitive test scores. Maternal and child health journal.

[16] Berkman DS, Lescano AG, Gilman RH, Lopez SL, Black MM (2002). Effects of stunting, diarrhoeal disease, and parasitic infection during infancy on cognition in late childhood: a follow-up study. The Lancet.

[17] Daniels MC, Adair LS (2004). Growth in young Filipino children predicts schooling trajectories through high school. The Journal of nutrition.

[18] Wechsler, D. *The measurement and appraisal of adult intelligence*. 4th ed. Baltimore: Williams & Wilkins (1958).

[19] Batty GD, Mortensen EL, Nybo Andersen AM, Osler M (2005). Childhood intelligence in relation to adult coronary heart disease and stroke risk: evidence from a Danish birth cohort study. Paediatric and perinatal epidemiology.

[20] Hart CL (2004). Childhood IQ and cardiovascular disease in adulthood: prospective observational study linking the Scottish Mental Survey 1932 and the Midspan studies. Social science & medicine.

[21] Zeng L (2008). Impact of micronutrient supplementation during pregnancy on birth weight, duration of gestation, and perinatal mortality in rural western China: double blind cluster randomised controlled trial. BMJ.

[22] Li C (2015). Prenatal micronutrient supplementation is not associated with intellectual development of young school-aged children. The Journal of nutrition.

[23] Li Q (2009). Effects of maternal multimicronutrient supplementation on the mental development of infants in rural western China: follow-up evaluation of a double-blind, randomized, controlled trial. Pediatrics.

[24] Anderson JW, Johnstone BM, Remley DT (1999). Breast-feeding and cognitive development: a meta-analysis. The American journal of clinical nutrition.

[25] Carpenter DO (2001). Effects of metals on the nervous system of humans and animals. International journal of occupational medicine and environmental health.

[26] Fattah C (2010). Maternal weight and body composition in the first trimester of pregnancy. Acta obstetricia et gynecologica Scandinavica.

[27] World Health Organization. Obesity: preventing and managing the global epidemic. World Health Organization (2000). 11234459

[28] Onis MD (2007). Development of a WHO growth reference for school-aged children and adolescents. Bulletin of the World health Organization.

[29] Chen H, Keith TZ, Weiss L, Zhu J, Li Y (2010). Testing for multigroup invariance of second-order WISC-IV structure across China, Hong Kong, Macau, and Taiwan. Personality and Individual Differences.

[30] Wechsler, D. *Manual for the Wechsler Intelligence Scale for Children*. 4th ed. San Antonio: The Psychological Corporation (2003).

[31] Weiss, L. G., Saklofske, D. H., Prifitera, A. & Holdnack, J. A. *WISC-IV advanced clinical interpretation*. Academic Press (2006).

[32] Filmer D, Pritchett LH (2001). Estimating wealth effects without expenditure data-or tears: an application to educational enrollments in states of India. Demography.

[33] Li C (2016). Sex differences in the intellectual functioning of early school-aged children in rural China. BMC public health.

[34] Christian P (2010). Prenatal micronutrient supplementation and intellectual and motor function in early school-aged children in Nepal. JAMA.

[35] Zhou J (2016). Maternal Prenatal Nutrition and Birth Outcomes on Malnutrition among 7-to 10-Year-Old Children: A 10-Year Follow-Up. The Journal of pediatrics.

[36] Adair LS (2013). Associations of linear growth and relative weight gain during early life with adult health and human capital in countries of low and middle income: findings from five birth cohort studies. The Lancet.

[37] Igel U, Romppel M, Baar J, Brähler E, Grande G (2016). Association between parental socio-economic status and childhood weight status and the role of urbanicity. Public health.

[38] Neggers YH, Goldenberg RL, Ramey SL, Cliver SP (2003). Maternal prepregnancy body mass index and psychomotor development in children. Acta obstetricia et gynecologica Scandinavica.

[39] Keim SA, Pruitt NT (2012). Gestational weight gain and child cognitive development. International journal of epidemiology.

[40] Institute of Medicine and National Research Council. Weight Gain During Pregnancy: Reexamining the Guidelines. The National Acadamines Press: Washington DC (2009). 20669500

[41] Kramer MS (2002). Breastfeeding and infant growth: biology or bias?. Pediatrics.

[42] Ong KK, Emmett PM, Noble S, Ness A, Dunger DB (2006). Dietary energy intake at the age of 4 months predicts postnatal weight gain and childhood body mass index. Pediatrics.

[43] Deary, I. J., Strand, S., Smith, P. & Femandes, C. Intelligence and educational achievement. *Intelligence*35, 13-21 (2007).

