# Supplementary material for: Effect of maternal pre-pregnancy underweight and average gestational weight gain on physical growth and intellectual development of early school-aged children
Source: Sci Rep. 2018 Aug 13;8:12014. doi: 10.1038/s41598-018-30514-6 (PMC6089877; doi:10.1038/s41598-018-30514-6)
Supplement: Supplementary file 1 — Supplementary table 1 Baseline characteristics comparison between follow-up and lost to follow-up groups [file 41598_2018_30514_MOESM1_ESM.doc]

| **Effect of maternal pre-pregnancy underweight and average gestational weight gain on physical growth and intellectual development of early school-aged children**  Chao Li ^1^, Ni Zhu ^2^, Lingxia Zeng ^1^, Shaonong Dang ^1^, Jing Zhou ^1^, Leilei Pei ^1^, Victoria Watson ^3^, Tao Chen ^3^, Duolao Wang ^3^ & Hong Yan ^1 4 5*^  ^1^ Department of Epidemiology and Biostatistics, School of Public Health, Xi’an Jiaotong University Health Science Center, Xi’an, China; ^2^ Department of Health Information, Shaanxi Provincial Center for Disease Control and Prevention, Xi’an, China; ^3^ Department of Clinical Sciences, Liverpool School of Tropical Medicine, Pembroke Place, Liverpool, United Kingdom; ^4^ Nutrition and Food Safety Engineering Research Center of Shaanxi Province, Xi’an, China; ^5^ Key Laboratory of Environment and Genes Related to Diseases, Xi’an Jiaotong University, Xi’an, China.  ∗ Correspondence: Hong Yan, School of Public Health, Xi’an Jiaotong University Health Science Center, No. 76 West Yanta Road, PO Box 46, Xi’an 710061, Shaanxi, China (e-mail: [yh.paper_xjtu@aliyun.com](mailto:yh.paper_xjtu@aliyun.com)).  Tel: +86 29 82655001; Fax: +86 29 82655387  **Supplementary table 1**  Baseline characteristics comparison between follow-up and lost to follow-up groups ^1^ | | | |
| --- | --- | --- | --- |
|  | Groups | | *P* |
|  | Follow-up | Lost to follow-up |
| **Child characteristics** |  |  |  |
| Number of children | 1744 | 1121 |  |
| Child age, mean±SD, y | 8.8±0.83 | 8.8±0.78 | 0.14 |
| Birth weight, mean±SD, kg | 3.2±0.43 | 3.2±0.42 | 0.71 |
| Gender, n (%) |  |  | 0.10 |
| Boy | 1045(59.9) | 637(56.8) |  |
| Girl | 699(40.1) | 484(43.2) |  |
| Gestational age at birth, mean±SD, wk | 39.9±1.68 | 39.8±1.73 | 0.40 |
| **Women’s characteristics** |  |  |  |
| Maternal age, mean±SD, y | 34.0±4.51 | 34.1±4.37 | 0.59 |
| Women's education, n (%) |  |  | 0.44 |
| <3 years | 121(7.0) | 65(5.8) |  |
| Primary | 512(29.4) | 314(28.1) |  |
| Secondary | 909(52.2) | 614(54.9) |  |
| ≥ High school | 198(11.4) | 125(11.2) |  |
| Women's occupation at enrollment, n (%) |  |  | 0.72 |
| Farmer | 1517(87.4) | 969(87.0) |  |
| Others | 86(12.6) | 106(13.0) |  |
| BMI at enrollment, mean±SD | 20.9(2.21) | 20.9(2.32) | 0.41 |
| **Others** |  |  |  |
| Father's education, n (%) |  |  | 0.39 |
| <3 years | 28(1.6) | 17(1.5) |  |
| Primary | 260(15.0) | 142(12.7) |  |
| Secondary | 1096(63.0) | 725(64.7) |  |
| ≥ High school | 355(20.4) | 236(21.1) |  |
| Father's occupation at enrollment, n (%) |  |  | 0.19 |
| Farmer | 1408(80.1) | 884(76.5) |  |
| Others | 332(19.9) | 236(21.1) |  |
| Household wealth index at enrollment, n (%) |  |  | 0.67 |
| Poorest | 620(35.6) | 411(36.6) |  |
| Middle | 621(35.6) | 381(34.0) |  |
| Richest | 503(28.8) | 329(29.4) |  |

^1^ Values are n(%) or means ± SD
